# Sex-specific cortical networks drive social behavior differences in an autism spectrum disorder model

**DOI:** 10.1038/s41398-025-03464-7

**Published:** 2025-07-21

**Authors:** Mariana Lapo Pais, José Sereno, Vanessa A. Tomé, Carla Fonseca, Camila Seco, Inês Ribeiro, João Martins, Ana Fortuna, Antero Abrunhosa, Luísa Pinto, Miguel Castelo-Branco, Joana Gonçalves

**Affiliations:** 1 University of Coimbra, Faculty of Sciences and Technology, Coimbra, Portugal; 2 University of Coimbra, Coimbra Institute for Biomedical Imaging and Translational Research (CIBIT), Coimbra, Portugal; 3 University of Coimbra, Institute for Nuclear Sciences Applied to Health (ICNAS), Coimbra, Portugal; 4 University of Coimbra, CQC-IMS, Chemistry Department, Coimbra, Portugal; 5 ICNAS Pharma, University of Coimbra, Coimbra, Portugal; 6 University of Coimbra, Laboratory of Pharmacology, Faculty of Pharmacy, Coimbra, Portugal; 7 Life and Health Sciences Research Institute (ICVS), School of Medicine, University of Minho, Braga, Portugal; 8 ICVS/3B’s - PT Government Associate Laboratory, Braga/Guimarães, Portugal; 9 University of Coimbra, Institute of Physiology, Faculty of Medicine, Coimbra, Portugal

**Keywords:** Molecular neuroscience, Autism spectrum disorders

## Abstract

Social behavior is highly sensitive to brain network dysfunction caused by neuropsychiatric conditions like autism spectrum disorders (ASDs). Some studies suggest that autistic females show fewer social skill impairments than autistic males. However, the relationship between sex differences in social behavior and sexually dimorphic brain neurophysiology in ASD remains unclear. We hypothesize that sex-specific changes in cortical neurophysiology drive the sexual dimorphism observed in social behavior for ASD. To test this, we used male and female *Tsc2*^*+/−*^ mice, a genetic ASD model, to examine cortical neuron morphology, the serotonergic system, E/I balance, structural connectivity, and social behavior. At the cellular level, transgenic males had shorter and less complex cortical basal dendrites, while transgenic females showed the opposite in apical dendrites. Notably, only *Tsc2*^*+/−*^ females exhibited changes in the serotonergic system and E/I balance, with reduced cortical 5-HT_1A_ receptor density and increased excitability. Additionally, activation of these serotonin receptors in transgenic animals correlated with E/I imbalance, highlighting inherent sexual dimorphisms in neuronal connectivity. In parallel, the TSC2 mouse model displayed sex-dependent changes in the structural connectivity of the cortex-amygdala-hippocampus circuit and social behavior: females showed a reduced number of axonal fiber pathways and reduced sociability, while males exhibited a loss of tissue density and deficits in social novelty. Moreover, in our ASD mouse model, better social performance correlated with the cortical serotonergic system. Our findings suggest that sex-dependent alterations in cortical neurophysiology, particularly in the serotonergic system, may contribute to the sexually dimorphic social behaviors observed in ASD.

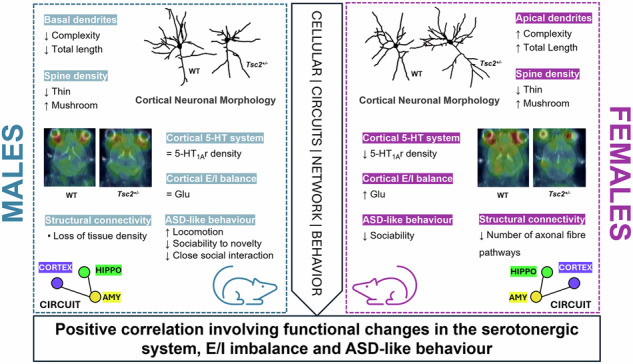

## Introduction

Autism spectrum disorder (ASD) is a neurodevelopmental condition characterized by social communication difficulties, restricted and repetitive behaviour and sensory anomalies [1] that affects 1 in 36 children [2]. This disorder is frequently diagnosed in males, with evidence suggesting that females are more likely to be misdiagnosed or underdiagnosed [3] and to show significantly better social interaction and communication skills, often referred to as social camouflaging [4, 5]. Despite the strong sexual dimorphism in social behavior for ASD, specific cellular and molecular alterations that might contribute to those distinct manifestations remain unclear.

The imbalance between excitatory and inhibitory (E/I) signaling is the most accepted hypothesis as a mechanism underlying social impairment in ASD [6]. Recently, ASD preclinical and clinical studies have provided evidence that E/I imbalance is heterogeneous both at the intra- and inter-subject [7–9]. Accordingly, it was observed that elevated excitatory activity, specifically in the mouse prefrontal cortex (PFC), results in impaired social behavior [10]. These findings indicated that biological sex interferes with social neuronal networks, especially E/I balance, leading to distinct autistic manifestations. In parallel, the serotoninergic (5-HTergic) system has also been recognized as a critical player in shaping social responses [11]. Actually, high 5-HT levels are associated with increased sensitivity to social factors [12]. Accordingly, enhancing 5-HT activity reverses impairments in sociability across multiple mouse models for ASD [13]. Strangely, despite well-known differences in the 5-HT signalling between males and females, including the rate of synthesis and receptor binding [14], sex as a biological variable is not included in most studies. Importantly, a study showed that although the 5-HTergic system significantly regulates social behavior in both males and females, its influence is more pronounced in females, particularly under stress-like conditions [15]. However, the role of the 5-HTergic system and E/I balance in the sex-specific social deficits observed between males and females with ASD remains poorly understood.

At the cellular level, both E/I balance and 5-HTergic are preserved through precise synaptic connections [16, 17]. The inherent morphology of neurons, including dendritic arborisations and dendritic spines, stands as a pivotal determinant for neuronal connectivity [18, 19]. The neocortex circuitry is organised to allow associative processing by comparing local inputs and long-range inputs [20], making it crucial to characterize abnormalities in both basal (integrate inputs from local circuits) and apical (typically process signals from distant brain regions) dendrites to establish a more comprehensive understanding of neural functions [20, 21]. Alterations in both morphological and molecular signaling can disrupt the typical development of structural and functional neural networks, potentially contributing to the clinical manifestations of some neuropsychiatric diseases [22]. In fact, data on distinct psychiatric disorders often linked symptoms such as social and cognitive impairments and emotional dysregulation to dysfunction within the cortex-hippocampal network [23–26]. Further, this overlap has also been suggested to be influenced by the network’s sensitivity to stress and its strong connection with the amygdala [26]. Based on that evidence, we hypothesized that cortical neurophysiological alterations in ASD can lead to network impairments in structural connectivity involving the cortex-amygdala-hippocampus circuit, which might be implicated in the pathophysiology of TSC.

At last, numerous pieces of convergent evidence collected from studies of humans and rodents suggested that the PFC plays a pivotal role in social interactions [23–25]. Accordingly, frontal cortical activity, explicitly involving the serotoninergic system, is crucial for regulating social, cognitive, and emotional functions [27, 28] as inhibitory (GABAergic) and excitatory (glutamatergic) transmission [29]. The distinct differences in ASD-related social skills between males and females underscore the critical need to explore the sexually dimorphic mechanisms underlying their molecular basis. Based on all the above-mentioned evidence, we hypothesized that cortical morphological and neurophysiological alterations linked to the serotonergic system may impact structural connectivity and be pivotal in the social impairments seen in ASD. By addressing this gap, our study could provide valuable insights into the sex-specific manifestations of ASD.

## Materials and methods

### Animals

**Fig. 1:**
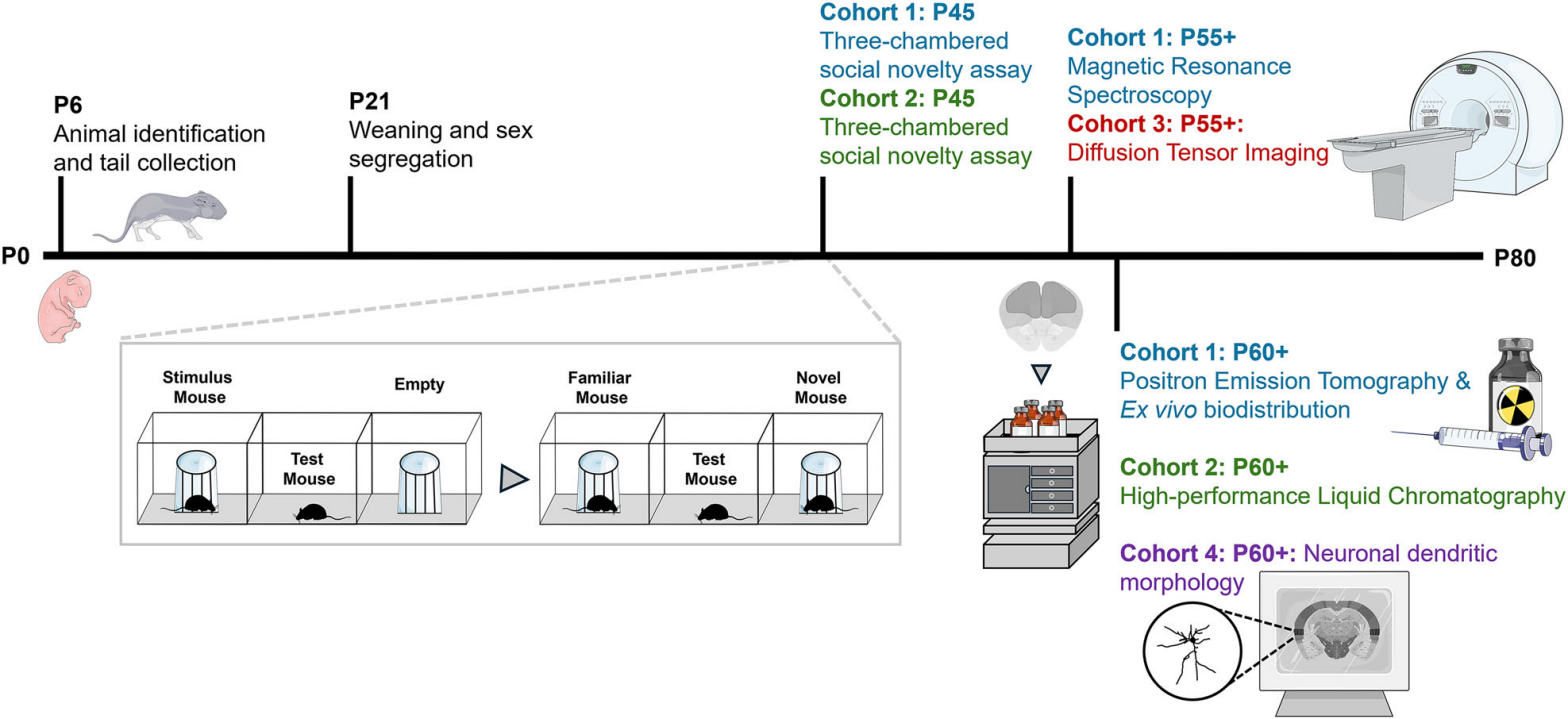
Timeline of all experiments and different cohorts in this study. Animals were identified on P6, and tail tips were collected for posterior genotyping. All pups were housed together with the dam until P21 and segregated by sex from P21 onwards. The three-chamber social novelty assay behavioral test was conducted at P45 for a dataset of cohorts 1 and 2 (*n* = 12 mice per group). A dataset of cohort 1 undergone in vivo ^1^H-MRS (P55 + ; *n* = 9–12 mice per group), in vivo PET imaging (P60 + ; *n* = 4–5 mice per group) and ex vivo biodistribution (P60 + ; *n* = 7–8 mice per group). In parallel, a dataset of cohort 2 underwent HPLC analysis (P60 + ; *n* = 13–15 mice per group). Lastly, in vivo DTI (P55 + ; *n* = 10–14 mice per group) and Golgi staining for neuronal morphological analyses (P60 + ; *n* = 4–5 mice per group) were conducted for cohort 3 and 4. ^1^H-MRS Proton Magnetic Resonance Spectroscopy, HPLC High-Performance Liquid Chromatography, DTI Diffusion Tensor Imaging, P Postnatal day, PET Positron Emission Tomography. The figure was partly generated using Allen Mouse Brain Atlas, atlas.brain-map.org, and Servier Medical Art, provided by Servier, licensed under a Creative Commons Attribution 3.0 unported license.

Heterozygous *Tsc2*^*+/−*^ mice with C57BL/6N background were crossed with C57BL/6J wild-type (WT) mice to generate experimental animals as previously described [30]. The *Tsc2*^*+/+*^ littermates were used as controls and referred to as wild-type (WT). Mice were housed at the Institute of Nuclear Science Applied to Health (ICNAS) animal facility at 22 °C under a 12-h light-dark cycle and provided ad libitum access to food and water. All experimental procedures were reviewed and approved by the Animal Welfare and Ethics Body of ICNAS (8/2023) following the guidelines of the European Community for the use of animals in the laboratory (86/609/EE) and the Portuguese law for the care and use of experimental animals (DL n° 129/92). A timeline of all experiments and different cohorts is represented in Fig. 1. All experiments were conducted and analyzed by an operator blinded to sex and genotype, with no randomization applied.

### Neuronal dendritic morphology

#### Golgi-cox staining

Mice were anaesthetized intraperitoneally (i.p.) with a mixture of ketamine (200 mg/Kg) and xylazine (16 mg/Kg), followed by intracardiac perfusion with 0.9% sodium chloride (NaCl) solution (pH 7.3). Brains were carefully removed, immersed in Golgi-Cox solution, and kept in the dark for 15 days. Next, they were transferred to a 30% sucrose solution and kept in the refrigerator in the dark for 2–5 days. Coronal sections (200 µm) were obtained in a vibratome (Leica VT100S, Germany), collected in 6% sucrose and blotted dry onto gelatin-coated microscope slides. Brain sections were subsequently alkalinised in 18.7% ammonia, developed in Dektol (Kodak), fixed in Kodak Rapid Fix, dehydrated and xylene cleared before coverslipping.

#### Morphological analysis

Cortical regions were delineated based on the Allen Mouse Brain Atlas (atlas.brain-map.org) and pyramidal neurons were selected based on their cytoarchitecture, including a clear identifiable axon and apical dendrite. Dendritic arbors of selected neurons were traced at 100x (oil) magnification using a motorised microscope (BX51, Olympus) and Neurolucida software (Microbrightfield Version 2022, MBF Bioscience) with the AutoNeuron extension module. For each animal, 10 neurons were reconstructed, and a three-dimensional analysis of the reconstructed neurons was performed. Spines were studied in segments of 30 µm in the apical branch and classified into spine type. Spine percentages were calculated as (number of spines per type)/(total number of spines).

### In vivo imaging studies

Animals were anaesthetised using 1.5–2% isoflurane during acquisitions. Magnetic resonance (MR) and positron emission tomography (PET) acquisitions were performed on a 9.4T MR preclinical scanner (Bruker Biospec, Billerica MA) operated with ParaVision (v6.0.1) and on a MicroPET scanner based on resistive plate chamber detectors (RPC-PET) [31], respectively. Detailed description of MR imaging data acquisition methodology can be found in the Supplementary File.

### ^1^H-magnetic resonance spectroscopy

For localized proton magnetic resonance spectroscopy (^1^H-MRS), data were collected in a volume of interest placed on the prefrontal cortex (PFC). Spectra analysis was performed using linear combination modeling LCModel (Stephen Provencher Inc., Toronto, ON, Canada) [32]. Metabolite quantification was performed by applying the internal water reference method. The Cramer–Rao lower bounds (CRLB) under 20% for glutamate and 30% for γ-aminobutyric acid (GABA) were used as a reliability measure of the metabolite concentration estimation [33].

### [carbonyl-^11^C]WAY-100635 positron emission tomography

[*carbonyl*-^11^C]WAY-100635 was synthesized according to a published method described in [34], with some modifications to fit into the commercial radiosynthesis modules housed at ICNAS Pharma Unipessoal, Lda (Coimbra, Portugal). Detailed [*carbonyl*-^11^C]WAY-100635 radiosynthesis description can be found in the Supplementary File.

#### Acquisition

The radiotracer, [carbonyl-^11^C]WAY-100635 PET, a selective 5-HT_1A_ antagonist radiotracer, was administered intravenously (i.v.) in a total injected activity of ~10 uCi/g. Approximately 5 min after tracer injection, a 50-min list mode data was initiated. The bed had fiducial marks installed to align the PET image with Magnetic resonance imaging (MRI).

#### Quantification

Quantitative imaging was performed using PMOD (PMOD, v3.6; PMOD Technologies, Zürich, Switzerland, RRID:SCR_016547). PET images were co-registered with the anatomical MRI, and a volume of interest (VOI) including the whole cortex was used based on an integrated anatomical ATLAS (Ma-Benveniste-Mirrione) [35]. The percentage of injected dose per VOI volume (%ID/mL) was calculated as [[total positron (β+ radioactivity) concentration in the VOI (Bq/mL)]/[total positron (β+ radioactivity) injected (Bq)]*100] from 30–50 min post-injection. Additionally, the standardised uptake value (SUV) was calculated as [[total positron (β+ radioactivity) concentration in the VOI (Bq/mL)]/[total positron (β+ radioactivity) injected (Bq)/body weight (g)]] from 30‐50 min post-injection. The SUV ratio (SUVr) is calculated by dividing the SUV in the target region by the SUV in the cerebellum (reference region).

### Ex vivo biodistribution of [carbonyl-^11^C]WAY-100635

Animals were injected with [carbonyl-^11^C]WAY-100635 intravenously (i.v.) in a total injected activity of ~10 uCi/g. After 50 min, a cardiac perfusion with saline solution was done to clean out all circulating blood, and ex vivo biodistribution (percentage of injected dose per tissue weight, %ID/g of tissue) was performed by gamma counting (CRC® – 55 tW radioisotope dose calibrator, Capintec, Ramsey, NJ, USA) in the PFC.

### High-performance liquid chromatography

Animals were euthanized, and PFC samples were collected and stored at −80 °C. Tissues were homogenized in ice-cold 0.2 M perchloric acid with 6 mM Cysteine and centrifuged for 15 min at 4147 × g at 4 °C. The supernatant was then deproteinised with 2.0 M perchloric acid and centrifuged for 4 min at 20817 × g at 4 °C. Following tissue preparation, tryptophan (tryp) and 5-HT were quantified with a Shimadzu high-performance liquid chromatography (HPLC) system (Shimadzu Corporation, Kyoto, Japan), constituted by a solvent delivery unit (LC-20A), a degasser system (DGU-20A5), an autosampler (SIL-20AHT), a column oven (CTO-10ASVP) and a fluorescence detector ((RF-20AXS). Chromatographic separations were performed based on the previous validated method [36].

### Diffusion tensor imaging

Each diffusion tensor image (DTI) acquisition consisted of 22 slices, 0.5 mm thick, encompassing the entire brain. DTI data were post-processed using TrackVis and Diffusion Toolkit (Ruopeng Wang, Van J. Wedeen, TrackVis.org, Martinos Center for Biomedical Imaging, Massachusetts General Hospital). For each acquisition, an anatomical ATLAS including whole cortex, amygdala and hippocampus was generated from the anatomical MRI and using PMOD (PMOD, v3.6; PMOD Technologies, Zürich, Switzerland, RRID:SCR_016547) integrated anatomical ATLAS, Mouse (Ma-Benveniste-Mirrione) [35]. Tract-based analysis was performed for the cortex, amygdala and hippocampus to obtain the total fibers number, the fractional anisotropy (FA) and the apparent diffusion coefficient (ADC) values.

### Three-chambered social novelty test

Each test animal was placed in the three-chamber test arena [37] in a red-light environment (7 LUX). The assay consisted of two phases, the first to study social interaction and the second to study social novel interaction, after a 10-min habituation period with two empty wire cages (Empty) in the side chambers.

- Sociability test phase (first phase): a cage with an unfamiliar mouse (social stimulus) was introduced to one side, and the test mouse was allowed to explore all three chambers freely for 10 min.
- Social novelty test phase (second phase): the empty wire cage was replaced with a cage with another unfamiliar mouse (novel stimulus). The subject was allowed to explore all three chambers for 10 min.

Unfamiliar mice were age- and sex-matched and previously habituated to the wire cages. Locomotory metrics were examined from top-video recordings using the Tracking (SMART) video system (v2.5; Panlab S.L., Spain). Total time sniffing social and novel stimulus was manually quantified. The social and social novel indexes for each animal were respectively calculated as [Social index = ((Social-Empty))/((Social + Empty))] and [Social Novel index = ((Novel-Familiar))/((Novel + Familiar))], each variable referring to the total time sniffing the respective stimulus.

### Statistical analysis

Sample size was defined to ensure adequate power within acceptable limits while minimizing animal use according to [E = Total number of animals – Total number of groups; any sample size, which keeps E between 10 and 20 was considered as adequate] [38]. Statistical analyses were performed using GraphPad Prism version 8.0.1 (GraphPad Software, San Diego, California) and R studio (Integrated Development Environment for R. Posit Software, PBC, Boston, MA). Results are presented as mean values ± standard error of mean (SEM), with similar variance between groups. All datasets were tested for normality with the Shapiro–Wilk test. For non-parametric data, quantile-quantile (Q-Q) plots were evaluated, and parametric tests were used as observations lay approximately on the straight line of the Q-Q plot. Analyses of variance (ANOVA) with Sidak’s test were used for multiple comparisons. The Pearson correlation, or the non-parametric alternative Spearman correlation, was computed to assess the relationship between multiple variables. Three-dimensional Sholl analysis was used to evaluate the spatial arrangement of dendritic material by quantifying the number of dendritic intersections at concentric 10-μm intervals from the soma by 2WAY ANOVA repeated measures with Sidak’s test for multiple comparisons. Outliers were removed using the ROUT method (Q = 5%).

## Results

### *Tsc2*^*+/−*^ animals exhibit sexual-dependent alterations in cortical neurons, affecting both basal and apical dendrites

**Fig. 2:**
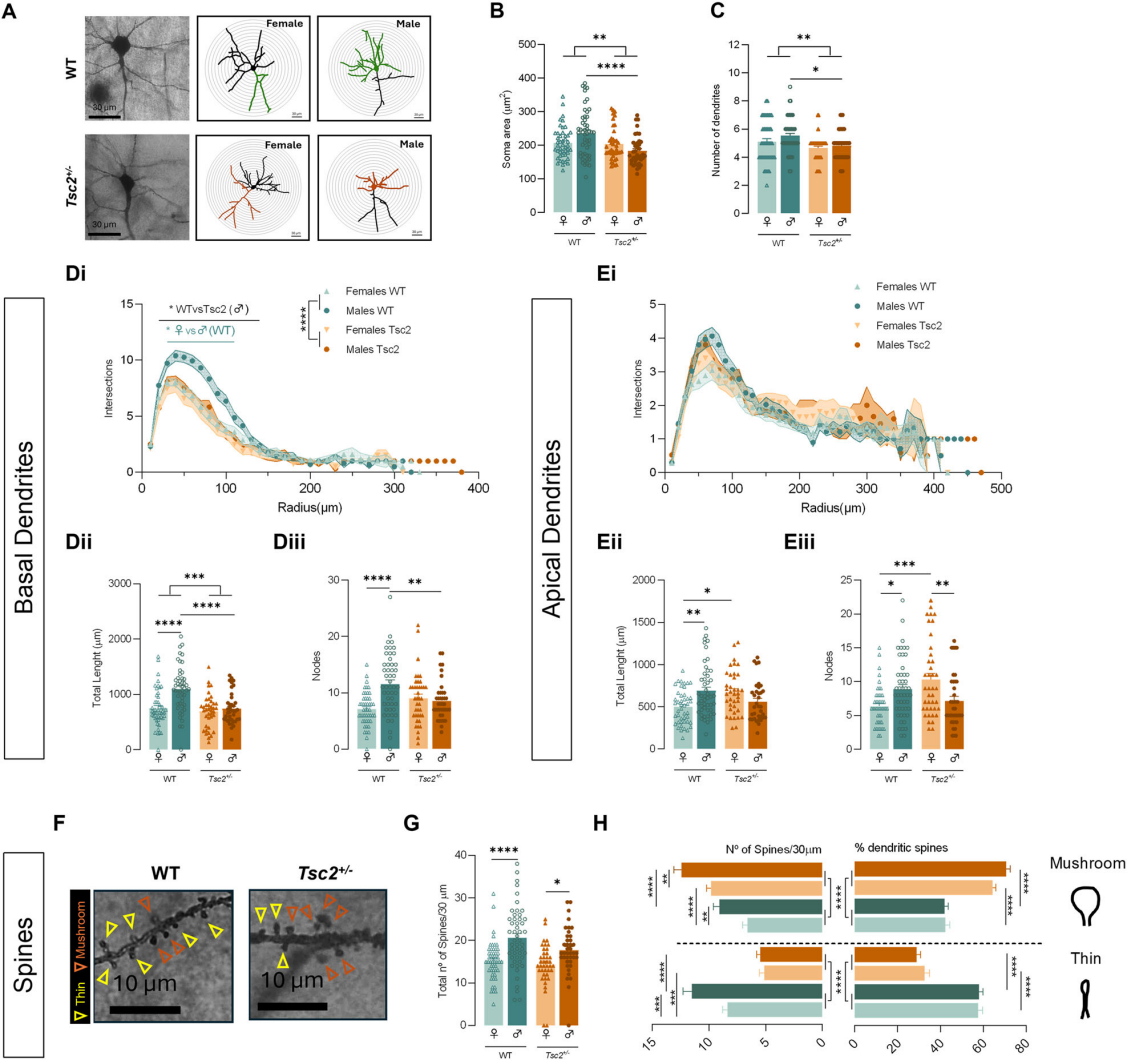
*Tsc2*^*+/−*^ animals exhibit sexual-dependent alterations in cortical neurons for both basal and apical dendrites. **A** Representative images of cortical pyramidal neurons labelled with Golgi-Cox and corresponding 3D reconstructions with soma and basal dendrites and apical dendrites highlighted with colour (green for WT and orange for *Tsc2*^*+/−*^) for males and females, respectively. Several aspects of dendritic morphology were examined, including soma area (µ*m*^2^) **B** and number of dendrites **C** for each genotype and sex. Moreover, basal dendrites intersections **Di**, total length (µm) **Dii** and nodes **Diii** and apical dendrites intersections **Ei**, total length (µm) **Eii** and nodes **Eiii** were accessed separately. **F** Representative images of dendritic spines of cortical neurons labelled with Golgi-Cox for WT and *Tsc2*^*+/−*^ mice. **G** The total number of spines was quantified in segments of 30 µm. Spines in the selected segments were classified into spine type, mushroom (top) and thin (bottom) and evaluated according to their **H**, left total number and **H**, right relative percentage. Data plotted as individual neuron values (dots) and mean ± SEM (columns and bars). Number of cells: 35–50 in *N* = 4–5 mice. *p < 0.05, **p < 0.01, ***p < 0.001, ****p < 0.0001 by 2WAY ANOVA with Sidak’s multiple comparisons-test **B,** **C**; **Dii**-**Diii**; **Eii**-**Eiii**; **G,** **H**. Three-dimensional Sholl analysis was used to evaluate the spatial arrangement of dendritic material by quantifying the number of dendritic intersections at concentric 10-μm intervals from the soma by 2WAY repeated measures ANOVA with Sidak’s multiple comparisons-test **Di**; **Ei**. The figure was partly generated using Allen Mouse Brain Atlas, atlas.brain-map.org.

Neuronal morphology and complexity of cortical dendritic arborization were assessed as indirect indicators of connectivity at the cellular level (Fig. 2). We reveal that genotype significantly influenced relevant neuronal morphological measures (soma area: F(1, 171) = 11.09, *p* < 0.01, Fig. 2B; number of dendrites: F(1, 174) = 10.13, *p* < 0.01, Fig. 2C), which can impact synaptic connectivity in *Tsc2*^*+/−*^ animals. Moreover, we exposed a significant interaction between genotype and sex (soma area: F(1, 171) = 8.906, *p* < 0.01, Fig. 2B) and sex-dependent changes after the post hoc tests analysis. Specifically, we exposed that transgenic males exhibit neuronal changes reflected by reduced soma areas (*p* < 0.01, Fig. 2B) and decreased dendritic complexity (number of dendrites: *p* = 0.04, Fig. 2C) compared with WT group.

Then, we characterize basal and apical dendrites separately and exposed that only basal dendrites were significantly influenced by genotype (intersections: F(1, 176) = 18.96, *p* < 0.01, Fig. 2Di; total length: F(1, 173) = 15.84, *p* < 0.01, Fig. 2Dii) and sex (intersections: F(1, 176) = 11.00, *p* < 0.01, Fig. 2Di; total length: F(1, 173) = 14.60, *p* < 0.01, Fig. 2Dii; nodes: F(1, 173) = 9.691, *p* < 0.01, Fig. 2Diii). However, an interaction between genotype and sex on both basal and apical dendrites was reported (basal intersections: F(1, 176) = 7.120, *p* < 0.01, Fig. 2Di; total basal length: F(1, 173) = 8.646, *p* < 0.01, Fig. 2Dii; basal nodes: F(1, 173) = 15.07, *p* < 0.01, Fig. 2Diii; total apical length: F(1, 163) = 14.60, *p* < 0.01, Fig. 2Eii; apical nodes: F(1, 171) = 18.88, *p* < 0.01, Fig. 2Eiii). Moreover, post hoc tests showed that basal arbors from transgenic male mice were less complex (intersections specifically proximal to soma: *p* < 0.05, Fig. 2Di; nodes: *p* < 0.01, Fig. 2Diii) and shorter (total length: *p* < 0.01, Fig. 2Dii) compared with WT males. In contrast, we showed that the apical dendritic arbors of transgenic females occupied higher regions (total length: *p* = 0.01, Fig. 2Eii) and displayed increased complexity (nodes: *p* < 0.01, Fig. 2Eiii) than those of WT females.

Furthermore, several sexual dimorphisms were reported specifically for control group. WT males presented increased dendritic complexity (basal intersections proximal to soma: *p* < 0.05, Fig. 2Di; basal nodes: *p* < 0.01, Fig. 2Diii; apical nodes: *p* = 0.02, Fig. 2Eiii) and larger arbor sizes (total basal length: *p* < 0.01, Fig. 2Dii; total apical length: *p* < 0.01, Fig. 2Eii) than WT females. In contrast, for transgenic mice, *Tsc2*^*+/−*^ males exhibited less complex apical dendrites compared with transgenic females (nodes: *p* < 0.01, Fig. 2Eiii).

Finally, to further evaluate synaptic connectivity, spines were quantified (Fig. 2F–H). Our results exposed that number of spines were only significantly influenced by sex (F(1, 171) = 32.81, *p* < 0.01, Fig. 2G) and not by genotype. In specific, males exhibited a higher number of spines compared with females in both transgenic (*p* = 0.0487, Fig. 2G) and control (*p* < 0.01, Fig. 2G) groups. Segregation by mushroom (mature) and thin (immature) spines revealed a significant effect of genotype on spine number (Thin: F(1, 170) = 66.86, *p* < 0.01; Mushroom: F(1, 174) = 42.58, *p* < 0.01, Fig. 2H) and their relative percentage (Thin: F(1, 170) = 177.3, *p* < 0.01; Mushroom: F(1, 166) = 179.0, *p* < 0.01, Fig. 2H). Moreover, we reported sexual dependent changes in spine number, reflect by a significant effect of sex (Thin: sex- F(1, 170) = 9.688, *p* < 0.01; Mushroom: sex- F(1, 174) = 25.63, *p* < 0.01, Fig. 2H) and interaction between genotype and sex (Thin: F(1, 170) = 6.031, *p* = 0.02, Fig. 2H) for this measure. Post hoc tests indicated that transgenic male and female mice exhibited lower number and relative percentage of thin spines (*p* < 0.01, Fig. 2H) but higher number and relative percentage of mushroom spines (*p* < 0.01, Fig. 2H) comparing to their correspondent control group. Moreover, sex-specific alterations were consistent across both WT and transgenic groups, with male mice exhibiting a higher number of spines compared to females (*p* < 0.01, Fig. 2H).

### Sex-specific changes in the cortical serotonergic system were strongly correlated with glutamate concentrations in *Tsc2*^*+/−*^ mice

**Fig. 3:**
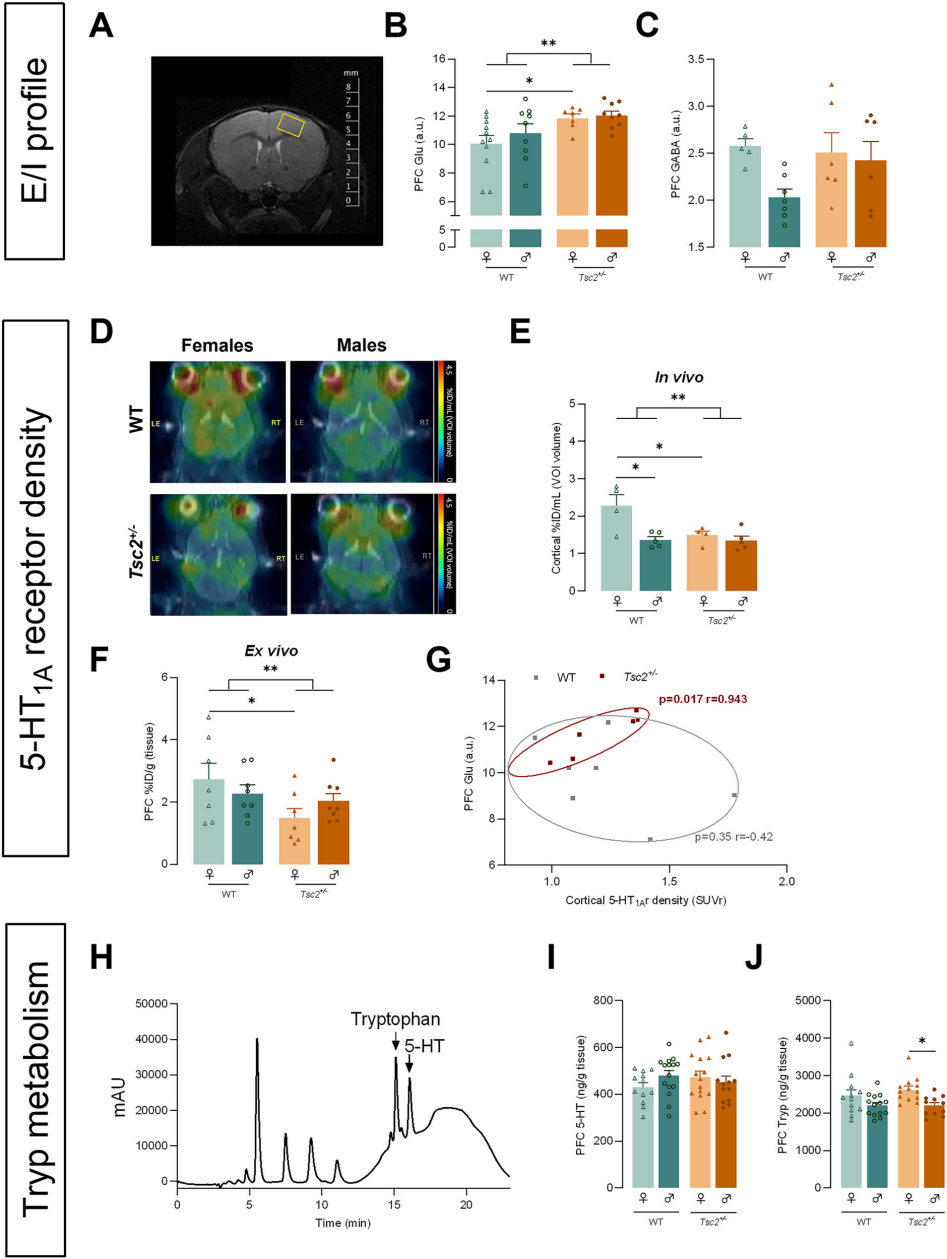
Sex-specific changes in the cortical serotonergic system were strongly correlated with glutamate concentrations in *Tsc2*^*+/−*^ mice. **A** Representative MRI image with MRS voxel (yellow square) localisation in the PFC. E/I balance was accessed from **B** in vivo PFC Glu concentration (a.u.) and **C** GABA concentration (a.u.) for each genotype and sex. **D** Representative MRI/PET in vivo neuroimage of [carbonyl-^11^C]WAY-100635. 5-HT_1A_ receptor density was accessed from **E** in vivo cortical %ID/g (body weight) and **F** ex vivo PFC %ID/g (tissue) for each genotype and sex. **G** Correlation between PFC Glu concentration (a.u.) and cortical 5-HT_1A_ receptor density (SUVr) for each genotype. **H** Representative chromatogram of PFC samples collected from control mice. PFC concentrations (ng/g tissue) of **I** 5-HT and **J** tryp for each genotype and sex. The results are expressed as mean ± SEM [*n* = 5–11 **B,** **C,** *n* = 4–5 **E,** *n* = 7–8 **F,** *n* = 6–7 **G,** *n* = 13–15 **I,** **J** for each group]. *p < 0.05, **p *<* *0.01* by 2WAY ANOVA with Sidak’s multiple comparisons-test **B,** **C,** **E**–**G,** **I,** **J** and Pearsons’s correlation (G). %ID/mL Percentage of Injected Dose per mL, 5-HT Serotonin, a.u. arbitrary units, Glu Glutamate, MRI Magnetic Resonance Imaging, MRS Magnetic Resonance Spectroscopy, PET Positron Emission Tomography, PFC Prefrontal Cortex, ROI Region Of Interest, Tryp tryptophan.

After assessing cortical neuronal morphology, we evaluate PFC E/I profiles using in vivo ^1^H-MRS (Fig. 3A–C). Cortical E/I profile analysis revealed a significant effect of genotype for glutamate concentrations (F(1, 32) = 8.35, *p* < 0.01, Fig. 3B) and post hoc tests exposed that only *Tsc2*^*+/−*^ females exhibited significantly higher excitability than their WT littermates (*p* < 0.05, Fig. 3B). For the GABA concentrations (Fig. 3C) and Glu/GABA ratio (Figure S1, Supplementary File), we did not report any significant effect on genotype or sex.

Then, functional changes in the serotonergic system, particularly 5-HT_1A_ receptor density, were assessed using in vivo neuroimaging with [carbonyl-^11^C]WAY-100635 (Fig. 3D, E). For [carbonyl-^11^C]WAY-100635 binding, we reported, once again, a significant influence of genotype, sex (genotype – F(1, 14) = 5.79, *p* = 0.03; sex - F(1, 14) = 9.767, *p* < 0.01, Fig. 3E) and their interaction (F(1, 14) = 5.235, *p* = 0.04, Fig. 3E). Post hoc analyses further revealed that only *Tsc2*^*+/−*^ female mice had lower cortical 5-HT_1A_ receptor density compared with WT group (*p* = 0.04, Fig. 3E). A sexual dimorphism for control animals was also reported reflecting an increased binding in females (*p* = 0.01, Fig. 3E). Considering that receptor binding is also coupled to the delivery of the radiotracer from the vascular space to the cellular target, we also performed ex vivo analysis after circulating blood clearance. From ex vivo biodistribution, only genotype significantly influenced the uptake of radioligand (F(1, 26) = 4.793, *p* = 0.04, Fig. 3F). According to the in vivo results, once again, only transgenic female mice had a significantly lower uptake of [carbonyl-^11^C]WAY-100635 compared with their WT littermates (*p* = 0.04, Fig. 3F). Since the same animals underwent to PET imaging and ^1^H-MRS [WT: *n* = 7 (3 females and 4 males); *Tsc2*^*+/−*^: *n* = 6 (3 females and 3 males)], we tested the association of these receptor and neurotransmitter measures (Fig. 3G) and found a strong positive correlation between cortical 5-HT_1A_ receptor density (SUVr) and glutamate concentrations (Spearman correlation; *p* = 0.02; r = 0.943, Fig. 3G) only in *Tsc2*^*+/−*^ animals.

Next, we analyze 5-HT levels and its biochemical precursor, tryptophan, in the PFC using HPLC, providing reliable indicators of 5-HT dysfunction and substrate availability for synthesis (Fig. 3H–J). Interestingly, our analysis of PFC revealed that while control and transgenic mice had similar 5-HT levels for both sexes (Fig. 3I), it was observed that sex significantly influenced tryp concentrations (F(1, 46) = 10.19, *p* < 0.01, Fig. 3J). *Tsc2*^*+/−*^ female mice presented significantly higher tryp levels than transgenic males from post hoc test analysis (*p* < 0.05, Fig. 3J).

### *Tsc2*^*+/−*^ mice exhibited altered structural connectivity involving the cortex-amygdala-hippocampus circuit in a sex-dependent matter

**Fig. 4:**
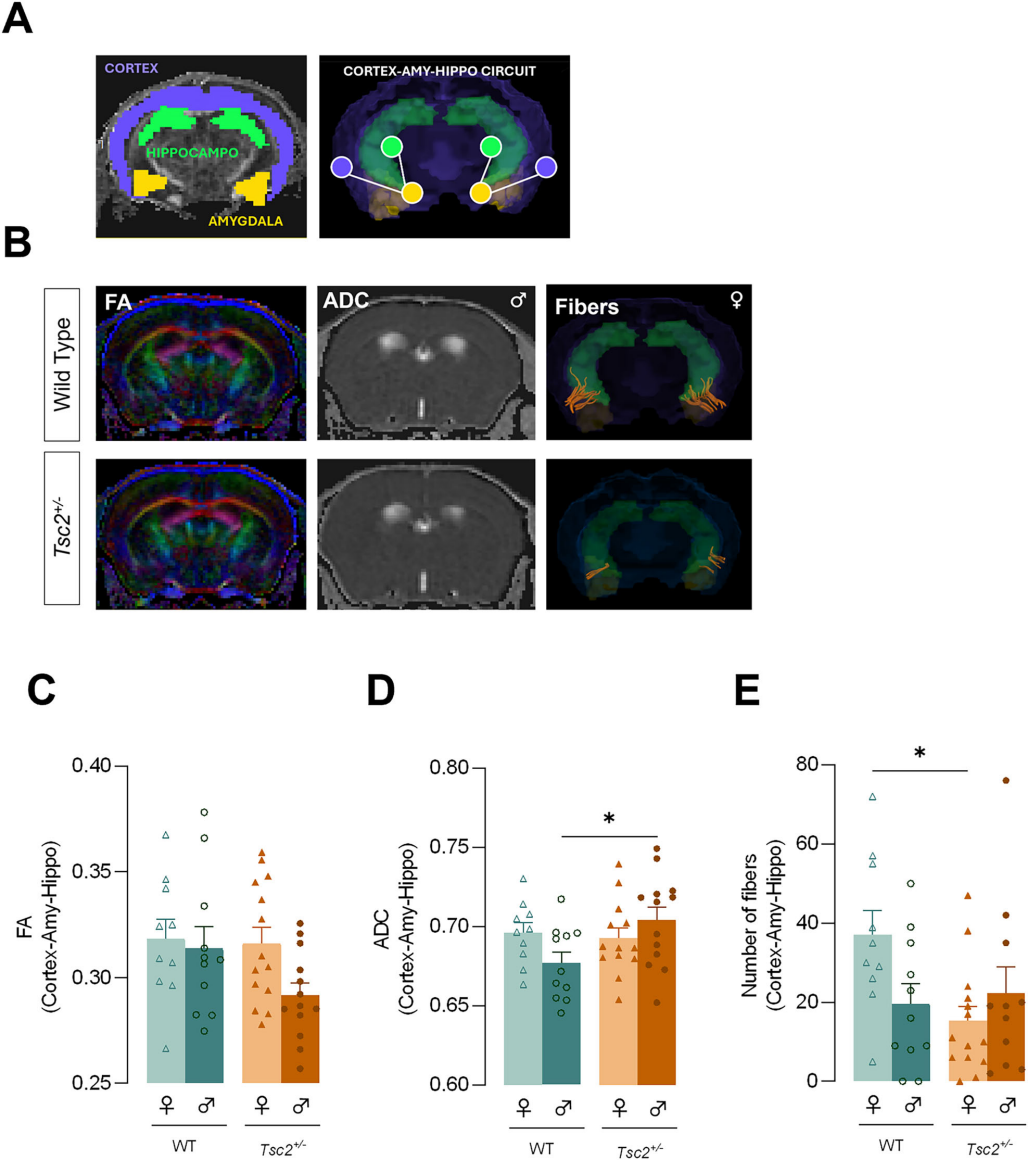
*Tsc2*^*+/−*^ mice exhibited altered structural connectivity involving the cortex-amygdala-hippocampus circuit in a sex-dependent matter. **A** Representative ROI masks for the whole cortex (purple), amygdala (yellow) and hippocampus (green) and a schematic diagram of the cortex-amygdala-hippocampus circuit. **B** Representative images for both WT and *Tsc2*^*+/−*^ mice of FA color maps, ADC images, and fiber tracts from the sex with the most reported alterations. Structural connectivity measures included **C** FA and **D** ADC values and **E** the number of fiber tracts within the cortex-amygdala-hippocampus circuit. The results are expressed as mean ± SEM (*n* = 10–14 for each group). *p < 0.05 by 2WAY ANOVA with Sidak’s multiple comparisons test. ADC Apparent diffusion coefficient, AMY Amygdala, FA Fractional anisotropy, HIPPO Hippocampus, ROI Region of interest.

Given that morphological changes may compromise brain structure and communication between regions, we explored potential alterations in structural connectivity between the cortex and other key networks, such as the amygdala and hippocampus, linked to the pathophysiology of several psychiatric diseases [26] (Fig. 4). Our analysis of structural connectivity for the cortex-amygdala-hippocampus circuity revealed a significant interaction between genotype and sex in apparent diffusion coefficient (ADC) values (F(1, 43) = 4.579, *p* = 0.0381, Fig. 4D) and the number of fibers (F(1, 42) = 5.299, *p* = 0.0264, Fig. 4E). Interestingly, *Tsc2*^*+/−*^ male mice presented significantly higher ADC values than WT males (*p* = 0.0438, Fig. 4D), while transgenic females exhibited fewer fibers than their control littermates (*p* = 0.0266, Fig. 4E).

### *Tsc2*^*+/−*^ mice exhibited sex-specific social deficits strongly correlated with tryptophan metabolism

**Fig. 5:**
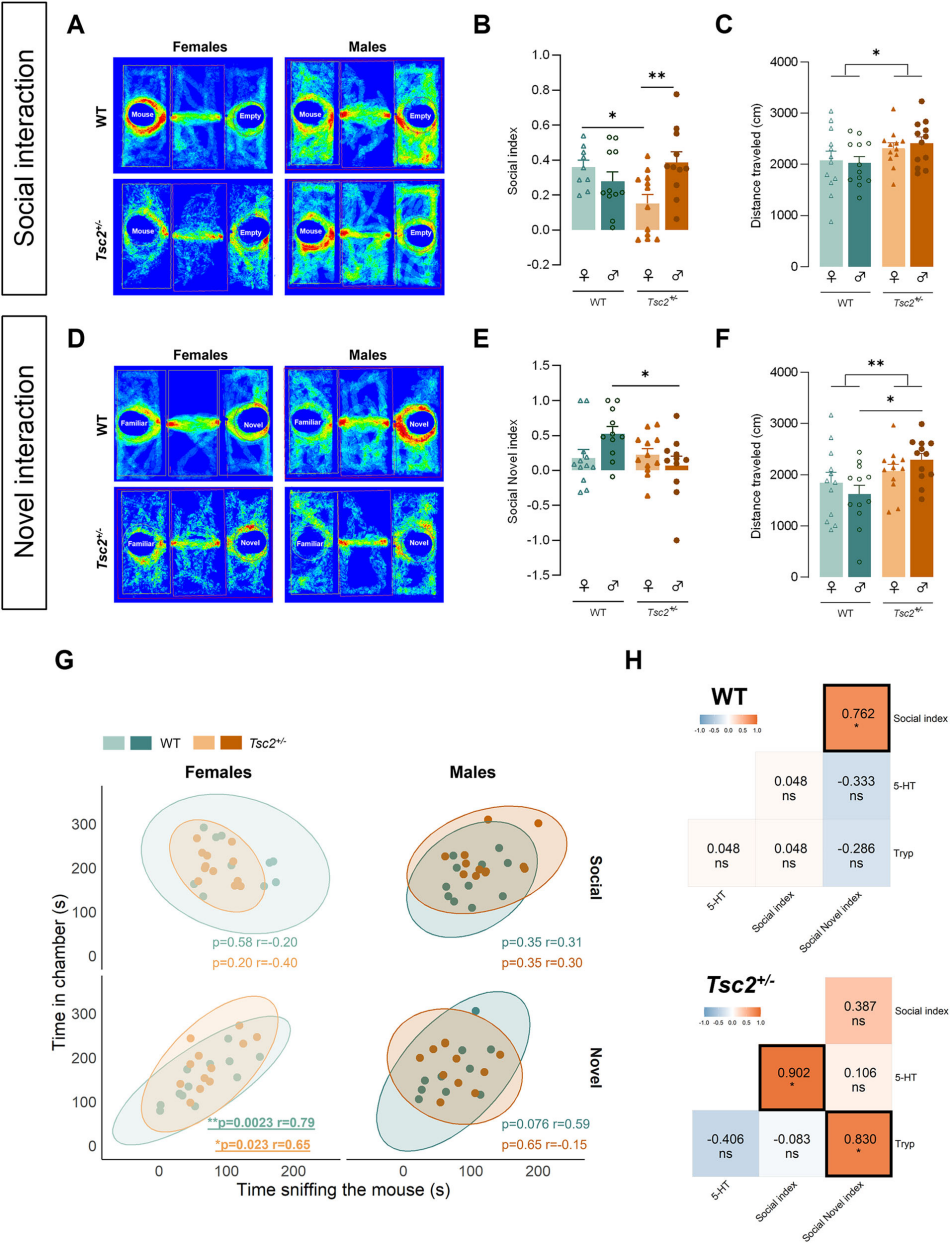
*Tsc2*^*+/−*^ mice exhibit sex-dependent impairment in social behavior strongly correlated with Tryp and 5-HT PFC levels. **A,** **D** Representative locomotory images for the sociability and social novelty test phases. Behavioral measures were examined for sociability phase, including **B** social index and **C** total distance traveled (cm) and social novelty phase, including **E** social novel index and **F** total distance traveled (cm) for each genotype and sex. **G** Correlations between time spent in the social/novel chamber (s) and time spent sniffing the social/novel mouse (s) were performed for each sex and genotype. Moreover, correlations between **H** social behavior and PFC 5-HT and tryp levels were accessed for (top) WT and (bottom) transgenic mice. The results are expressed as mean ± SEM (*n* = 9–12 **B,** **C,** **E**–**G,** *n* = 6–8 **H** for each group). *p < 0.05, **p *<* *0.01* by 2WAY ANOVA with Sidak’s multiple comparisons-test **B,** **C,** **E,** **F**, Spearman’s correlation (**H**, WT) and Pearsons’s correlation (**G,** **H,** *Tsc2*^*+/−*^).

To investigate social interaction, a three-chambered social novelty test was performed (Fig. 5). From both phases we found a significant interaction between genotype and sex (social index: F(1, 39) = 8.724, *p* < 0.01, Fig. 5B; social novel index: F(1, 42) = 4.889, *p* = 0.03, Fig. 5E) and a significant influence of genotype for the total distance traveled (sociability phase: F(1, 44) = 4.980, *p* = 0.03, Fig. 5C; social novelty phase: F(1, 44) = 7.624, *p* < 0.01, Fig. 5F).

Post hoc test analysis further revealed that, transgenic females had higher social deficits during first phase while both locomotory and social alterations were particularly noticeable among transgenic males during second phase. Specifically, *Tsc2*^*+/−*^ females had a lower social index compared with WT females (*p* = 0.02, Fig. 5B) and *Tsc2*^*+/−*^ males (*p* < 0.01, Fig. 5B). Moreover, only transgenic male mice displayed lower social novelty preference for the novel stimulus (*p* = 0.05, Fig. 5E) and increased total distance traveled (*p* = 0.01, Fig. 5F), compared with their WT littermates. Interestingly, for all groups, time spent in the social chamber was not significantly correlated with time spent sniffing the social stimulus (Pearson correlation, Fig. 5G), which can be attributed to the unfamiliarity with the environment. In contrast, the time spent in the social novel chamber was significantly positively correlated with the time spent sniffing the novel stimulus in WT females (Pearson correlation, *p* < 0.01, r = 0.79, Fig. 5G), and showed a trend toward significance in WT males (Pearson correlation, *p* = 0.08, r = 0.59, Fig. 5G). In *Tsc2*^*+/−*^ mice, a significant positive correlation was observed only in females (Pearson correlation, *p* = 0.02, r = 0.65, Fig. 5G).

Correlation analysis between social behavior and HPLC data (WT: *n* = 8; *Tsc2*^*+/−*^: *n* = 6) showed that 5-HT and tryp concentrations were positively correlated with social index and social novel index in transgenic animals, respectively (Pearson correlation, social index: *p* = 0.014, r = 0.90; social novel index: *p* = 0.04, r = 0.83; Fig. 5H). Correlation analysis between social behavior and MRS data was also performed, but no significant correlations were found (Figure S1, Supplementary File).

## Discussion

Building on the hypothesis that sex-dependent changes in cortical morphology and neurophysiology contribute to distinct social impairments in males and females with ASD, we provide evidence that sexually dimorphic traits, ranging from cellular to neuronal circuit levels, may underlie network dysfunctions and the distinct social behavior patterns observed between sexes in ASD.

This study is the first to analyse basal and apical dendrite morphology in both males and females for the *Tsc2*^*+/−*^ mice, a mouse model exhibiting both ASD-like behavior and increased excitability [30]. Our results suggest that transgenic males have a limited capacity to integrate information from local circuits within the cortical region, implied by shorter and less complex basal dendrites. In females, apical dendrites might have developed in a more complex and longer morphology that could be a compensatory mechanism to maintain or enhance neural connectivity in response to information from distant brain regions. Accordingly, data on ASD and epilepsy [18, 39–42], mostly obtained in males, reported the same alterations that we observed in *Tsc2*^*+/*−^ males. These results unveil, for the first time, inherent sexual dimorphism in neuronal morphology within the context of ASD, potentially elucidating the pronounced sex differences in incidence and manifestations observed in this disorder. Dendritic spines are also an important aspect of neuronal morphology, strongly influencing neural network activity [43]. Another important finding of this work was that *Tsc2*^*+/−*^ animals have increased mushroom and reduced thin dendritic spine density in cortical pyramidal neurons. In agreement, a study reported that sub-threshold synaptic stimulation and activation of the mammalian target of the rapamycin (mTORC1), which results from *Tsc2* gene mutation [44, 45], resulted in significant enlargement of spine heads, a sign of enhanced excitatory synapse formation [46]. However, for ASD an increase in spine densities with immature morphology has been described, indicating a general spine immaturity state [40, 47]. From our results we can postulate that in this mouse model spines were adapted as a result of increased neural activity to establish enhanced excitatory synapse formation. Since brain circuits are preserved through precise synaptic connections [16], these cortical morphological alterations observed could originate changes in cortical neuronal transmission.

Additionally, here, we demonstrated that transgenic mice exhibited reduced 5-HT_1A_ receptor density that have been also described from neuroimaging and postmortem data in ASD patients [48–50]. In parallel, *Tsc2*^*+/−*^ mice had higher cortical concentrations of glutamate which agree with elevation in glutamatergic activity previously reported for *Tsc2*^*+/−*^ and ASD [30, 51]. Significantly, we found a female sex bias in those specific alterations as only *Tsc2*^*+/−*^ females exhibited higher excitability but reduced 5-HT_1A_ receptor density than their WT littermates. Importantly, it has been proposed that serotonin, acting on 5-HT_1A_ receptors, suppresses glutamate release [52, 53]. Yet, in our transgenic mice, we showed that greater cortical 5-HT_1A_ receptor density correlates with higher excitability. We argue that activation of 5-HT_1A_ receptor could be a result from higher excitability in *Tsc2*^*+/−*^ mice to compensate for that imbalance. However, from our results, that inhibitory effect was still insufficient to suppress glutamate release. From that, we postulate that serotonergic dysfunctions could be associated with the E/I imbalance shifted to excitability in this animal model and specifically for females. In addition, considering that GABA is unchanged this further supports a direct effect on glutamate transmission. Additionally, a sexual dimorphism in concentrations of tryp, a precursor of 5-HT, was also observed. Considering that *Tsc2*^*+/−*^ females exhibited significantly lower receptor density but comparable tryp and 5-HT levels than their WT littermates, we proposed that the presynaptic input remains intact, and the observed effects in *Tsc2*^*+/−*^ females are likely due to alterations in the postsynaptic 5-HT_1A_ receptor density on glutamatergic neurons. The fact that increased excitability correlates with higher 5-HT_1A_ receptor density in transgenic mice may reflect a dysregulation in the receptor signaling rather than a change in presynaptic function.

Alterations in both morphological and molecular signaling can disrupt the typical development of structural and functional neural networks [22]. Here, we also exposed that the TSC2 mouse model exhibited sex-dependent changes in structural connectivity, particularly in the cortex-amygdala-hippocampus circuit. Transgenic females showed a reduced number of axonal fiber pathways, while *Tsc2*^*+/−*^ males displayed a loss of tissue density reflected by increased ADC values, both indicating reduced microstructural integrity of white matter tracts, which has been previously reported in TSC [54–57]. Regarding sex-specific alterations, a previous study including only male patients reported increased diffusion values [58], consistent with our findings in *Tsc2*^*+/−*^ males. From our results, we propose that the sex-specific dysfunctions in neuronal morphology are directly linked to similar sex-dependent impairments in structural connectivity. In females, the reduced number of long-range axonal fibers (white matter tracts) likely affects axonal integrity and the neurons’ ability to transmit signals across distant regions. That could be linked to the apical dendritic alterations observed specifically in females. In males, the loss of tissue density likely reflects a reduced concentration of cells and cellular structures, which may result from smaller and less complex neurons reported in males. Importantly, the cortex-amygdala-hippocampus network is often implicated in social and cognitive impairments, emotional dysregulation, and stress-related disorders [23–26]. We believe that these sex-related network impairments may partially explain the sexually dimorphic symptoms in tuberous sclerosis complex (TSC) and related clinical manifestations, such as ASD [59].

Social interaction and communication deficits are widely recognised as one of the core ASD features [60], which are also observed in our animal model. In parallel, consistent research also suggests that there are significant differences between genders in people diagnosed with ASD [5, 61]. Our study indicated that *Tsc2*^*+/−*^ females had reduced sociability. We believe that exposure to a non-familiar animal for the first time could generate a more anxious condition in transgenic females, leading them to avoid social interaction. In a previous work, we hypothesized that social impairment in female *Tsc2*^*+/−*^ mice might represent a stress-coping strategy [30], potentially linked to the higher social anxiety observed in females, which is often associated with camouflaging behavior [5, 62]. On the other hand, only transgenic males showed increased locomotion and lower social novelty preference compared with their WT littermates, which agrees with previous work in other ASD mouse model [5]. Moreover, the transgenic males were the experimental group that faced more difficulties during close social interaction from the social novelty test phase. Interestingly, the females of our animal model resemble girls’ ability to camouflage their autistic traits seen in humans [5], since *Tcs2*^*+/−*^ females presented “normative” social patterns upon familiarisation. Importantly, for the first time, our data proved that cortical serotoninergic metabolism strongly correlates with social deficits in *Tcs2*^*+/−*^ mice. We showed that animals with higher cortical 5-HT and tryp concentrations had more developed social skills. This finding is aligned with previous studies indicating that systemic enhancement of the serotoninergic system reverses social deficits in multiple mouse models for ASD [13] and that increasing dietary tryp intake ameliorated autism symptoms [63].

It has been suggested that TSC-related neural alterations may underlie its clinical manifestations, potentially linking disruptions in cellular and circuit signaling to the development of neurological and neuropsychiatric symptoms, such as ASD and cognitive/intellectual impairments [22, 55, 64]. Interestingly, our study exposed that, similar to social deficits, structural connectivity alterations reflect dysfunctional network communication, though in distinct domains. Females exhibit a reduced number of white matter fibers within the network, while in males, the number of fibers remains unchanged, but their density is significantly reduced. This evidence suggests that the number of fibers may play a more primordial role in sociability—impaired in females—whereas fiber density may act as a secondary player influencing novel social interactions, as seen in males. Moreover, we propose that these sex-dependent impairments in structural connectivity result from sex-specific dysfunctions in neuronal morphology. Notably, altered 5-HT_1A_ receptor density and hyperexcitability, which likely reflect compromised communication between excitatory (glutamatergic) neurons, were observed exclusively in females. From that we believe that both E/I balance and 5-HTergic have a greater impact on sociability rather than on novel social interactions, further highlighting the sexually dimorphic nature of these disruptions. Moreover, based on our results, tryp supplementation may offer a potential therapeutic strategy to address social deficits, an approach that has also garnered interest for other psychiatric and neurological disorders associated with disruptions in tryp and 5-HT metabolism [65]. Our findings further imply that while the core symptoms of ASD may appear similar, the pathways leading to these symptoms can vary significantly between sexes. This insight underscores the importance of considering sex as a critical factor in both the study and treatment of ASD.

## Supplementary information

Supplemental Material

## Data Availability

Data and materials will be made available on request.
