# Supplementary material for: Sex-specific cortical networks drive social behavior differences in an autism spectrum disorder model
Source: Transl Psychiatry. 2025 Jul 21;15:251. doi: 10.1038/s41398-025-03464-7 (PMC12279983; doi:10.1038/s41398-025-03464-7)
Supplement: Supplementary file 1 — Supplemental Material [file 41398_2025_3464_MOESM1_ESM.docx]

***Proton magnetic resonance spectroscopy (^1^H-MRS) – Detailed data acquisition methodology***

B0 map was acquired before spectroscopy, and shims were optimized through a MAPSHIM voxel. Spectra were acquired using a point-resolved spectroscopy (PRESS) sequence with outer volume suppression (OVS) and VAPOR water suppression. The following parameters were used: TR = 2500 ms, TE = 16.225 ms, number of averages = 720, 3 flip angles = 90°, 142°, 142°, bandwidth = 5000 Hz, number of acquired points = 2048, yielding a spectral resolution of 1.22 Hz/pt. Before each spectrum, an unsuppressed water spectrum was acquired at the same voxel location (TE = 16.225 ms, TR = 2500 ms, 16 averages, scanning time = 40 s).

We acknowledge that due to overlapping spectral peaks and the presence of other metabolites, MRS does not allow for the complete isolation of Glu and GABA signals. Consequently, the measured concentrations may include contributions from related compounds, and this limitation should be considered when interpreting the results.

***[carbonyl-^11^C]WAY-100635 radiosynthesis***

The ^11^C-carboxylation of the Grignard reagent and the subsequent ^11^C-acylation of WAY-100634 precursor were carried out on Synthra [^11^C]Choline® module (Synthra GmbH, Hamburg, Germany) supported by an IBA Synthera® Extension platform (IBA, Belgium). The product purification and solid phase extraction reformulation were performed on cassette-based synthesizer Trasis AllinOne (Trasis, Belgium). Quality control tests of the obtained [*carbonyl*-^11^C]WAY-100635, including appearance, pH, radiochemical identity, radiochemical purity, radionuclide identity, and residual solvent content were carried out following internal standard procedures. Analysis of radiochemical identity and radiochemical purity were performed on an analytical HPLC (Agilent 1260 Infinity II LC Series, Agilent, Santa Clara, CA, USA) equipped with radio and UV detection (270 nm) in series. A Zorbax® Eclipse XDB-C18 (80 Å, 150 × 4.6 mm, 5 µm) column was used, with an isocratic method of 0.1 M ammonium formate aqueous solution/Acetonitrile (50:50) and flow rate 2 mL/min. The retention time of [*carbonyl*-^11^C]WAY-100635 was approximately 4 min with radiochemical purity of 99%.

***Diffusion tensor imaging (DTI) – Detailed data acquisition methodology***

The following parameters were used: TR = 3000 ms and TE = 19.7806 ms. A HARDI scheme was used, and 120 diffusion sampling directions were acquired. The in-plane resolution was 0.125 mm. The b-value was 686.225 s/mm2, and an automatic quality control routine checked the b-table to ensure accuracy.

***Magnetic resonance imaging for co-registration of PET (positron emission tomography) and DTI (diffusion tensor imaging) studies***

T2-weighted images were acquired in coronal and axial planes using a RARE sequence: repetition time (TR) = 4181 ms; echo time (TE) = 33 ms; 5 averages; pixel size of 0.156 mm × 0.156 mm and slice thickness of 0.5 mm without spacing between slices (total head 256 pixels × 192 pixels × 40 slices).


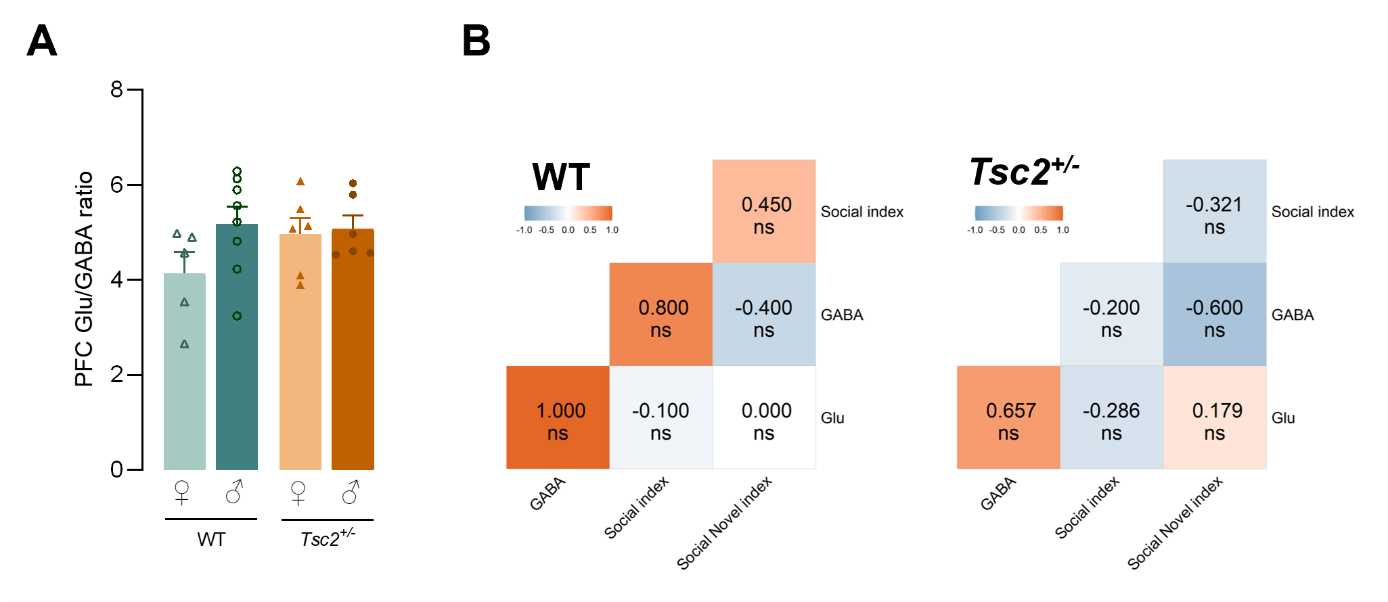


**Figure S1** – **Unchanged PFC Glu/GABA ratio and lack of correlation between E/I neurotransmitters and social behavior.** (A) *In vivo* PFC Glu/GABA ratio and (B) correlation between PFC E/I neurotransmitters [glutamate and GABA concentration (a.u.)] and social metrics (social index and social novel index). The results are expressed as mean ± SEM [n = 5-8 (A) and n= 4-9 (B) for each group]. *p<0.05 by 2WAY ANOVA with Sidak's multiple comparisons-test (A) and Spearmen’s correlation (B). a.u. – arbitrary units; Glu – Glutamate; PFC – Prefrontal Cortex.
